# Analytical Methods and Application of Single-Cell and Single-Nucleus Transcriptomics in the Study of Ischemic Stroke

**DOI:** 10.3390/biom16071054

**Published:** 2026-07-18

**Authors:** Changqing Mu, Yuchuan Ding, Alexander Weiss, Sydni Rosenfeld, Fengwu Li, Xiaokun Geng

**Affiliations:** 1 Luhe Institute of Neuroscience, Capital Medical University, Beijing 101149, China; muchangqing@mail.ccmu.edu.cn (C.M.);; 2 Department of Neurology, Beijing Luhe Hospital, Capital Medical University, Beijing 101149, China; 3 Department of Neurosurgery, Wayne State University School of Medicine, Detroit, MI 48201, USA; yding@med.wayne.edu (Y.D.); sydni-rosenfeld@wayne.edu (S.R.)

**Keywords:** bioinformatics analysis, differential expression analysis, trajectory inference, transcription factor analysis, cell–cell communication analysis

## Abstract

Background: Ischemic stroke remains a leading cause of mortality and long-term disability worldwide, with complex and heterogeneous pathophysiological processes. Single-cell and single-nucleus RNA sequencing (sc/snRNA-seq) has been increasingly applied to investigate cellular heterogeneity at high resolutions. Methods: We systematically searched PubMed, Web of Science, and Embase to identify studies that applied sc/snRNA-seq in ischemic stroke research. Based on the retrieved literature, we summarized the bioinformatic analytical methods and application strategies reported in these studies, focusing on how sc/snRNA-seq has been utilized across different research contexts. Results: The application of sc/snRNA-seq in ischemic stroke has expanded rapidly across species and sample types. A wide range of downstream bioinformatic analyses have been employed, including clustering, differential expression analysis, trajectory inference, gene regulatory network analysis, and cell–cell communication analysis. These approaches have been applied to investigate diverse biological processes in ischemic stroke. In addition, these analytical strategies have been extended to multiple biological contexts, including extracerebral tissues, stroke-related modifiers, and their associated complications. Furthermore, integrative analytical approaches that combine multiple datasets, bulk transcriptomics, and other omics data have been increasingly utilized. Advances in temporal and spatial resolutions have enabled analyses across different stages and anatomical regions. Conclusions: This review systematically summarizes the analytical methods and application strategies of sc/snRNA-seq in ischemic stroke. These approaches provide a structured perspective for understanding the application of single-cell technologies in this field. Future studies may benefit from standardized designs and coordinated analytical strategies to facilitate more systematic investigations.

## 1. Introduction

Ischemic stroke remains a leading cause of adult mortality and long-term disability worldwide, with a growing global incidence and an increased disease burden [1,2]. Globally, there are over 7.8 million new ischemic strokes each year, and by 2030, the number of cerebral infarctions worldwide is projected to increase by 1.76 million [3,4]. Despite significant progress in reperfusion therapy, the clinical translation of neuroprotective strategies has repeatedly failed, indicating a lack of understanding of the heterogeneity of cellular and molecular events after stroke [5]. There is a need for a more comprehensive and precise understanding of the pathophysiological mechanisms of ischemic stroke to identify novel therapeutic targets and intervention strategies.

**Figure 1:**
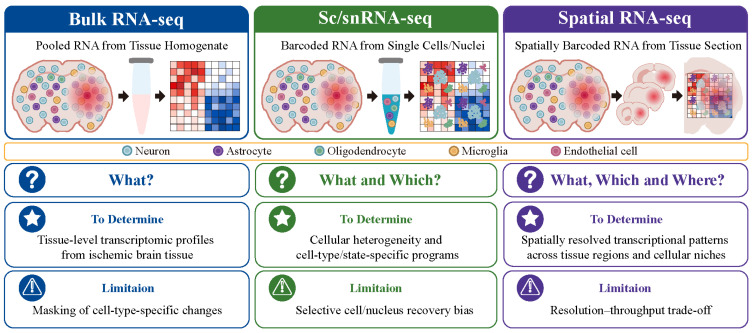
Comparison of Bulk RNA-seg, sc/snRNA-seg, and Spatial Transcriptomics. Bulk RNA-seq identifies overall gene-expression changes at the tissue level but masks cell-type-specific responses. Sc/snRNA-seq reveals both the molecular changes and the cell types or cellular states in which they occur, although selective cell or nucleus recovery may introduce bias. Spatial RNA-seq further preserves anatomical context, showing what changes, which cells are involved, and where these changes occur within ischemic regions and cellular niches, while balancing spatial resolution and data throughput. Its application remains influenced by platform-dependent trade-offs between spatial resolution and throughput.

Since its introduction in 2009, single-cell RNA sequencing (scRNA-seq) has advanced transcriptomic profiling and was recognized as the “Method of the Year” within five years of its introduction [6,7]. In the same year, single-nucleus RNA sequencing (snRNA-seq) was first demonstrated [8] and has since been widely applied in neuroscience research, particularly since 2016 onward [9,10,11]. Together, these technologies, collectively referred to as sc/snRNA-seq, enable the characterization of cellular heterogeneity at single-cell resolution [12]. In contrast to bulk RNA sequencing, which captures averaged transcriptomic signals across heterogeneous cell populations, sc/snRNA-seq allows gene expression analysis at the level of individual cells or nuclei (Figure 1) [13].

In recent years, these techniques have been increasingly applied in ischemic stroke research, providing additional perspectives for the investigation of complex biological processes. Recent reviews have summarized sc/snRNA-seq findings in ischemic stroke, mainly focusing on cell-type-specific responses, cellular heterogeneity, intercellular interactions, and related biological discoveries [14,15]. Building on these important studies, the present review takes a different and complementary approach. Rather than organizing the literature mainly by cell type, we structure the review around key stroke-related mechanisms and translational themes, including neuroinflammation, BBB disruption and angiogenesis, cell death, metabolic dysregulation, aging and senescence, neuroregeneration and remyelination, extra-central nervous system (CNS) responses, stroke complications, and therapeutic interventions. This framework highlights how sc/snRNA-seq has advanced mechanistic understanding of ischemic stroke and how these insights may support target discovery and therapeutic development.

In this review, we summarize the analytical methods and application strategies of sc/snRNA-seq in ischemic stroke, including their use in different research contexts and approaches for data integration and spatiotemporal analysis. This study aims to provide a reference for future studies.

## 2. Methods and Search Strategy

This review systematically summarizes the discoveries derived from single-cell and single-nucleus RNA sequencing (sc/snRNA-seq) studies related to ischemic stroke. We conducted a comprehensive literature search using the PubMed, Web of Science, and Embase databases, applying combinations of the following keywords: “single cell RNA sequencing”, “scRNA-seq”, “single cell transcriptome”, “single nucleus RNA sequencing”, “snRNA-seq”, and “single nucleus transcriptome” in conjunction with “ischemic stroke”, “brain ischemia”, “cerebral infarction”, and “stroke”. We included peer-reviewed articles and preprints published or publicly available until 22 December 2025.

The inclusion criteria were as follows: (1) original research articles or secondary analyses that utilized sc/snRNA-seq; (2) studies focusing on ischemic stroke in either human subjects or preclinical models; (3) reports that provided sufficient methodological details of sc/snRNA-seq analysis; and (4) publications written in English. We excluded (1) reviews, editorials, conference abstracts, letters, books, and (2) retracted publications or duplicate entries.

**Figure 2:**
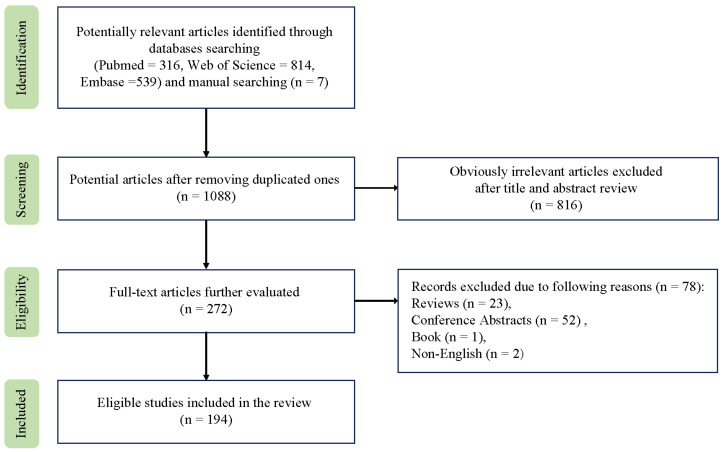
Flowchart Illustrating the Literature Search and Selection Process.

The initial search yielded 1669 articles through database searching and an additional seven articles through manual searching. After removing 588 duplicates and 816 irrelevant articles based on their titles/abstracts, 78 articles were excluded after full-text review because they were reviews, editorials, conference abstracts, case reports, books, letters, or clinical protocols or did not meet the inclusion criteria. Ultimately, 194 original studies met the inclusion criteria. The final selection process is illustrated in Figure 2.

## 3. Comparison of scRNA-Seq and snRNA-Seq

scRNA-seq profiles intact cells and captures both nuclear and cytoplasmic transcripts, generally yielding greater transcript complexity and more detected genes than snRNA-seq [16]. However, scRNA-seq typically requires fresh, viable cell suspensions and enzymatic or mechanical dissociation, which may preferentially deplete large or fragile neural cells and induce ex vivo stress or activation-associated transcriptional changes, particularly in microglia and other brain myeloid cells [17]. In contrast, snRNA-seq can be applied to frozen, archived, or difficult-to-dissociate brain tissues and may reduce dissociation-induced transcriptional artifacts [16,18,19]. Its main limitation is that nuclear RNA represents only a fraction of the whole-cell transcriptome and contains a higher proportion of intronic and nascent transcripts [16,20]. The two approaches may also recover different cell populations, with snRNA-seq potentially underrepresenting some immune cells [21]. Neither approach preserves spatial architecture by itself, but both can complement spatial transcriptomics by providing cell type-resolved reference profiles for interpreting spatial gene-expression patterns. Thus, scRNA-seq and snRNA-seq should be viewed as complementary rather than interchangeable approaches, and their selection should depend on sample availability, tissue characteristics, and the biological question.

## 4. Evolution of sc/snRNA-Seq Applications in Ischemic Stroke

**Figure 3:**
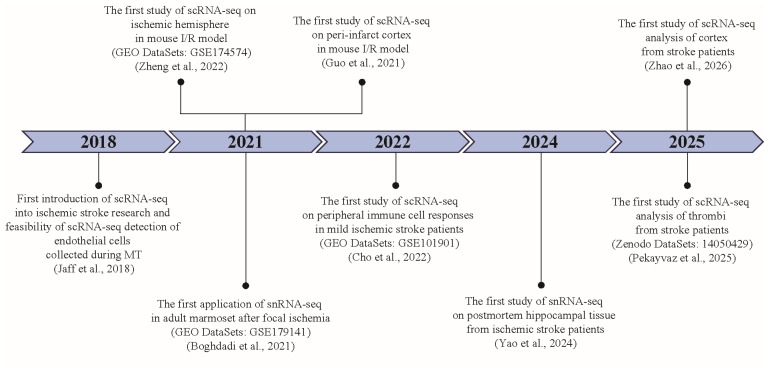
Development and Key Milestones of scRNA-seq and snRNA-seq Applications in Ischemic Stroke Research [22,23,24,25,26,27,28,29].

The application of sc/snRNA-seq technologies to ischemic stroke has expanded rapidly in recent years (Figure 3). This review references 194 articles that have applied sc/snRNA-seq technologies. Among these 194 references, 92 (47%) articles were published since 2025, reflecting the rapidly changing nature of this field. Key milestones illustrate this progression in the field. In 2018, scRNA-seq was first applied in stroke research [22]. In 2021, Zheng et al. and Guo et al. independently published the first scRNA-seq atlases of the ischemic hemisphere and peri-infarct cortex in a mouse ischemia/reperfusion (I/R) model [23,24]. In the same year, the first snRNA-seq dataset for stroke was generated using focal ischemia in adult marmosets [25]. Subsequent studies extended these approaches to human-derived samples, including peripheral blood in 2022 [26], postmortem hippocampal tissue in 2024 [27], and post-stroke cortex and thrombi in 2025 [28,29]. Additionally, sequencing data from Zheng et al. has become the most widely reanalyzed scRNA-seq dataset in the stroke field, serving as a benchmark for secondary analysis and method developments.

## 5. Analytical Framework and Evolving Applications of sc/snRNA-Seq in Ischemic Stroke

The standard workflow of sc/snRNA-seq has been extensively characterized in previous studies [30,31]. Briefly, the pipeline includes tissue dissociation, single-cell or single-nucleus isolation, mRNA capture, cDNA synthesis and amplification, library construction, high-throughput sequencing, and bioinformatics analysis. Analytical strategies for sc/snRNA-seq can be broadly grouped into preprocessing and downstream analysis.

### 5.1. Data Preprocessing

Preprocessing typically includes cell quality control, normalization, batch-effect correction, feature selection, and dimensionality reduction. Cells are filtered based on total unique molecular identifier (UMI) counts, number of detected genes, and the proportion of mitochondrial or ribosomal transcripts to exclude low-quality or artifactual cells [32,33]. Ambient RNA contamination can be minimized using tools such as SoupX [34], DecontX [35], CellBender [36], and scCDC [37]. Doublet detection and removal, commonly performed using DoubletFinder [38] and Scrublet [39], identifies and excludes doublet libraries generated when two or more cells are captured together and sequenced as one. If not removed, these technical artifacts may be mistaken for intermediate or transitional cell states and can confound clustering, visualization, and downstream analyses [40]. Gene expression is commonly normalized using Seurat log-normalization or SCTransform [41]. For batch correction and dataset integration, tools such as Seurat’s canonical correlation analysis (CCA)-based integration [42], Harmony [43], and scMerge [44] are frequently used. After integration, highly variable genes are selected for dimensionality reduction, usually through principal component analysis (PCA) [32].

Published ischemic stroke studies have used different preprocessing tools, filtering thresholds, and parameter settings [23,24,45]. The same public dataset may also be processed differently across original and secondary analyses [23,46]. This heterogeneity underscores the lack of stroke-specific preprocessing benchmarks and may limit direct comparisons across studies.

### 5.2. Downstream Analytical Strategies

Although these downstream analytical approaches differ computationally, they collectively address complementary biological questions. Clustering defines the cellular landscape after stroke, differential analyses identify molecular alterations within those cells, trajectory and regulatory analyses infer dynamic changes and upstream regulators, whereas cell–cell communication and phenotype-associated analyses extend these findings to tissue-level organization and disease mechanisms.

#### 5.2.1. Cell Clustering and Annotation

Downstream analyses typically begin with clustering and cell type annotation. Graph-based community detection methods, particularly Louvain and Leiden, are widely used for clustering scRNA-seq and snRNA-seq data [47]. Cell type annotation can be performed using classical marker genes or automated tools, including SingleR, scmap [48], and GPTCelltype [49]. In ischemic stroke, these analyses establish the cellular framework required to identify injury-associated microglia, infiltrating immune cells, neurovascular populations, and other disease-specific cellular states before downstream functional analyses can be performed. However, clustering is sensitive to preprocessing, feature selection, dimensionality reduction, and resolution parameters, which may merge or split dynamic post-stroke cell states [50]. This is particularly relevant for microglia, which exhibit inflammatory, interferon-responsive, proliferative, and phagocytic programs rather than discrete M1/M2 states [23,51]. Neutrophil subclustering is also limited by sparse recovery in whole-brain scRNA-seq datasets, making rare subsets vulnerable to sampling time, cell recovery, and clustering resolution [52,53].

#### 5.2.2. Cellular Composition and Differential Abundance

Differential abundance analysis quantifies changes in cell populations between conditions. Conventional methods, such as chi-squared tests, provide intuitive comparisons, whereas compositional approaches such as scCODA [54] better account for compositional bias in single-cell data. In addition, neighborhood-based methods such as Milo [55] improve the detection of continuous or transitional cell states without relying on rigid cluster boundaries. These analyses help quantify immune-cell infiltration, loss of vulnerable neuronal populations, expansion of reactive glia, and changes in vascular cell populations associated with tissue repair in stroke. However, inferred cell proportions reflect the relative composition retained after tissue sampling, isolation, capture, and quality control rather than absolute cell numbers in the original tissue [54]. Differences in cell type recovery across workflows, including between scRNA-seq and snRNA-seq, may therefore contribute to apparent abundance changes and complicate comparisons across studies [18,21].

#### 5.2.3. Differential Gene Expression

Differential expression (DE) analysis is widely used to identify genes and pathways altered between conditions. Cell-level methods such as the Wilcoxon rank-sum test are commonly used for exploratory analyses but typically treat cells as independent observations and may underestimate biological variability between samples [56,57]. In contrast, pseudobulk approaches using DESeq2 or edgeR aggregate counts at the sample level and provide more robust inference across biological replicates [57], while mixed-effects models offer an alternative [56,58]. In ischemic stroke, differential expression analyses identify cell-type-specific transcriptional responses underlying neuroinflammation, neuronal injury, angiogenesis, and tissue remodeling. Nevertheless, DE findings remain influenced by sample size, study design, and model assumptions and should be validated in independent biological samples.

#### 5.2.4. Functional Enrichment

Functional enrichment analysis is widely used to identify biological pathways perturbed after ischemia using gene sets such as Gene Ontology (GO) [59], Kyoto Encyclopedia of Genes and Genomes (KEGG) [60], and custom-defined signatures. Common approaches include over-representation analysis (ORA) [61], which tests enrichment among DEGs, and gene set enrichment analysis (GSEA) [62], which analyzes the entire ranked gene list to detect coordinated pathway-level changes. These analyses help determine which biological processes are preferentially activated in different cell populations after stroke, such as inflammatory signaling, oxidative stress, ferroptosis, or vascular remodeling. However, enrichment results are sensitive to analytical choices, including gene selection thresholds, ranking metrics, background gene sets, enrichment methods, and pathway databases, and may vary across parameter settings and knowledge resources [63,64,65]. Moreover, enrichment indicates statistical association rather than causal pathway activation and requires biological validation [65].

#### 5.2.5. Gene Set Scoring

Unlike differential expression, gene set scoring evaluates biological pathway activity at the single-cell level, allowing comparison of processes such as senescence, ferroptosis, and glycolytic activation across cell populations. Methods such as AddModuleScore, GSVA [66], ssGSEA, AUCell, and UCell [67] quantify the activity of predefined gene sets in each cell or sample, although their performance can be affected by gene set quality and data sparsity [68]. Gene set scores are also influenced by gene set definition, data characteristics, and algorithm selection; therefore, higher scores reflect stronger transcriptional signatures rather than necessarily indicating functional activation [68,69].

#### 5.2.6. Metabolic Pathway Inference

Metabolic pathway inference methods extend conventional gene set scoring by estimating metabolic activity or flux states at single-cell resolution. Tools such as scMetabolism [70] primarily quantify pathway activity using enrichment-based scoring strategies, whereas Compass [71] and scFEA [72] further incorporate metabolic network or flux-based modeling to infer functional metabolic states from transcriptomic data. Because ischemic injury profoundly alters cellular energy metabolism, these methods have become increasingly useful for investigating metabolic reprogramming in immune and vascular cells after stroke. However, these estimates should be interpreted cautiously because transcript-based metabolic inference does not directly measure enzyme activity or metabolite abundance. This limitation may be particularly relevant in ischemia/reperfusion, where metabolic states change rapidly and are sensitive to tissue handling [72,73,74,75].

#### 5.2.7. Trajectory and Cell Fate Inference

Trajectory inference methods reconstruct pseudotemporal cellular transitions from progressive transcriptional changes. Tools such as Monocle2 [76], Monocle3 [77], and Slingshot [78] infer lineage trajectories by ordering cells along continuous transcriptional states. RNA velocity [79] further estimates the direction of short-term transcriptional change from the relative abundance of spliced and unspliced transcripts, whereas CellRank [80] integrates RNA velocity with Markov chain-based transition probabilities to infer initial and terminal cell states. In addition, methods such as CytoTRACE [81] and scEntropy [82] estimate cellular differentiation potential, stemness, and transcriptional plasticity. In stroke, trajectory inference helps reconstruct activation and differentiation programs that cannot be directly observed experimentally, such as transitions from homeostatic to activated microglia or reparative immune phenotypes. However, inferred trajectories can be influenced by algorithmic assumptions, prior information, feature selection, dimensionality reduction, and parameter settings, and different analysis choices may yield different topologies or cellular orderings [83,84]. Pseudotime represents a computational ordering of transcriptional states rather than chronological time, and inferred trajectories do not independently establish lineage relationships or cell fate conversion.

#### 5.2.8. Transcription Factor and Gene Regulatory Network

Transcription factor (TF) activity and gene regulatory programs can be inferred using prior knowledge-based or data-driven approaches. DoRothEA provides confidence-ranked TF–target regulons for TF activity inference [85], whereas SCENIC constructs regulons from the analyzed scRNA-seq data through TF–gene co-expression and motif enrichment analyses [86]. These analyses help identify upstream regulators that may drive disease-associated transcriptional programs and represent potential therapeutic targets. However, these approaches provide indirect computational inference rather than evidence of direct TF binding or causal regulation, and their results may vary according to the selected regulon resource, data characteristics, and analytical choices [86,87,88].

#### 5.2.9. Cell–Cell Communication

Cell–cell communication (CCC) analysis infers potential intercellular signaling networks by modeling ligand–receptor interactions between sender and receiver cell populations. Tools such as CellChat [89], CellPhoneDB [90], and NicheNet [91] use distinct statistical frameworks and prior ligand–receptor resources to characterize intercellular crosstalk and prioritize biologically relevant signaling pathways. Because ischemic stroke involves coordinated responses among neurons, glia, vascular cells, and infiltrating immune cells, CCC analysis has become an important strategy for identifying signaling networks that coordinate injury and repair. However, CCC inference depends on curated ligand-receptor databases and method-specific algorithms, which may yield inconsistent results [92,93]. Moreover, ligand-receptor co-expression does not confirm functional signaling or spatial proximity and may miss metabolite- or neurotransmitter-mediated communication [94,95,96].

#### 5.2.10. Phenotype-Associated Cell and Gene Program

Several phenotype-associated analytical frameworks have been developed to link single-cell transcriptional features with clinical or experimental phenotypes. Methods such as Scissor [97] identify cell populations associated with specific external phenotypes, whereas hdWGCNA [98] detects phenotype-related co-expression gene modules across cells. Non-negative matrix factorization-based approaches, including consensus NMF (cNMF) [99] and geneNMF [100], further decompose transcriptional heterogeneity into latent gene programs that can be associated with pathological or functional states. These approaches prioritize cell populations and gene modules most closely associated with clinically relevant stroke phenotypes, thereby facilitating biomarker and therapeutic target discovery. However, these frameworks identify statistical associations rather than causal drivers, and their results may be influenced by phenotype quality, limited biological replication, confounding variables, model tuning, and dataset-specific structure. Therefore, selected cells, modules, or gene programs require replication in independent datasets [97,99].

#### 5.2.11. In Silico Perturbation

Emerging computational frameworks, such as scTenifoldKnk [101] and CellOracle [102], enable in silico perturbation analyses to predict how genetic perturbations may reshape cellular transcriptional states and regulatory networks. scTenifoldKnk simulates virtual gene knockout effects by comparing reconstructed gene regulatory networks before and after perturbation, whereas CellOracle further integrates single-cell transcriptomic and chromatin accessibility information to model transcription factor perturbations and their effects on cell states and regulatory programs. However, in silico predictions are constrained by the input data and the inferred regulatory network [101,102], and may show limited generalizability to unseen perturbations or cellular contexts [103]. In stroke research, these methods can prioritize candidate genes for experimental validation before labor-intensive knockout or pharmacologic studies are performed.

### 5.3. Application of sc/snRNA-Seq Bioinformatics Analysis in Ischemic Brain

Building on the methodological overview above, this section summarizes how downstream bioinformatic analyses have been applied in sc/snRNA-seq studies of ischemic stroke. These approaches have enabled cell atlas construction, comparison of cell-type and subset composition, identification of cell type-specific transcriptional programs, and inference of cell-state transitions, regulatory networks, and potential intercellular communication. In ischemic stroke, these analyses have provided cell-resolved insights into immune and inflammatory responses, neurovascular unit and blood–brain barrier-associated transcriptional alterations, angiogenesis, cell death, metabolic reprogramming, aging, neural regeneration, remyelination, and therapeutic responses. Applications of these downstream analytical strategies in ischemic stroke research are listed in Table 1.

Cell Type Analysis. Clustering, cell-type annotation, and cellular composition analysis provide the first layer of interpretation by defining the post-stroke cellular landscape. In an early representative study, scRNA-seq of mouse brain tissue collected 24 h after MCAO identified 17 major cell types and multiple microglia, CNS-associated macrophage, neutrophil, lymphocyte, endothelial, pericyte, and vascular smooth muscle cell subpopulations [23]. Composition analysis showed that monocyte-derived cells increased from approximately 2% to 16% after MCAO, with a relative increase in endothelial cells [23]. Additional studies identified expansion of the EC1 endothelial subset after ischemic stroke, decreased peri-infarct glutamatergic neurons on day 3 with partial recovery by day 14 [104,105], increased neural stem cells in the subventricular zone on day 3 [106]. Age-related comparisons further showed higher relative abundances of multiple immune cell populations in ischemic brain tissue from aged mice than from young mice at both 3 and 14 days after stroke [107]. Together, these analyses show how cellular composition analyses can define stroke-associated shifts across immune, vascular, and neural populations.

Differential Expression and Functional Enrichment Analysis. Differential expression and functional enrichment analyses link cell-resolved transcriptional changes to stroke-related mechanisms. Stroke-associated microglial subsets have shown enrichment for interferon-response or cell-cycle programs [23], whereas endothelial EC1 marker genes have been associated with vascular development and regulation of cell death [104]. Integration of sc/snRNA-seq data with bulk RNA-seq prioritized Cd72 and Cd74 as hub genes upregulated after stroke [108,109], while cell type-specific comparisons showed increased MMP9 and MMP12 expression in astrocytes after MCAO/R [110]. Gene deletion studies further localized molecular alterations to specific cell populations. Following Lrg1 deletion, adhesion-related genes were altered in BBB-associated cell populations with increased Ngf expression, and the corresponding DEGs were enriched for cell adhesion, oxidative stress defense, cell migration, hypoxia response, and cell death [111]. Following Nhe1 deletion, microglial and oligodendrocyte subsets displayed gene-expression features related to lipid metabolism, phagocytosis, and myelin support [112], while another Lrg1 deletion study identified enrichment in phagosome and oxidative phosphorylation pathways [113]. Age-stratified comparisons further showed increased Ifi27l2a expression in microglia from aged mice after stroke [107], and additional studies identified ferroptosis-related neuronal programs [114] and reduced cellular respiration and oxidative phosphorylation programs in microglia [115]. These findings illustrate how DE and enrichment analyses extend cell annotation toward mechanisms involving inflammation, neurovascular injury, aging, cell death, and metabolic dysfunction.

Gene Set Scoring to Evaluate Biological Signatures. Gene set scoring and metabolic inference provide complementary approaches for evaluating predefined pathological signatures and inferred metabolic activity. In ischemic stroke studies, gene set scoring has primarily been used to quantify immune activation, regulated cell death pathways (including ferroptosis, autophagy, and pyroptosis), metabolic programs, mitochondrial function, and cellular senescence [53,116,117,118,119,120,121,122,123,124,125]. Dedicated single-cell metabolic inference remains relatively more limited. scMetabolism analysis indicated that LCP1-high monocytes and macrophages after stroke exhibited transcriptionally inferred states related to fatty acid metabolism, biosynthesis, elongation, and degradation [126], whereas scFEA predicted reduced transport-module activity involving amino acid uptake in endothelial cells from ischemic stroke samples [127]. These approaches support cell type-resolved comparison of pathological signatures and inferred metabolic states, while remaining dependent on gene signatures or transcriptome-based metabolic models.

Trajectory Analysis for Cell-State Characterization. Trajectory analysis extends cell-state characterization by inferring potential transitions of molecular programs after stroke. In microglia, inferred trajectories have reconstructed transitions from homeostatic through intermediate to activated states during the acute phase [128]. At 90 days after MCAO, pseudotime analysis reconstructed a trajectory from homeostatic microglia to stroke-associated foamy microglia, with cholesterol metabolism-related genes upregulated earlier than inflammatory chemokine genes [129]. Age-related studies also showed that BrNeu2 neutrophils in aged ischemic brains were positioned toward the distal end of the inferred neutrophil trajectory and exhibited senescence- and inflammation-related features [130]. These analyses suggest that trajectory inference can help characterize stroke-associated cell-state transitions, the ordering of metabolic and inflammatory programs, and age-related shifts in immune states.

Cell–Cell Communication Analysis. Cell–cell communication analysis extends single-cell findings from individual cell populations to predicted intercellular signaling networks. Zheng et al. reported an increase in predicted interactions centered on microglia and CNS-associated macrophages after MCAO, involving pathways such as CCL, IL1, TNF, and SPP1 signaling [23]. In aged mice after stroke, predicted vascular- and myelin-repair-associated signaling between microglia/macrophages and endothelial or oligodendrocyte-lineage cells was broadly reduced, including VEGFA, GDF15, FN1, and SPP1 signaling [131]. These studies illustrate how communication analysis can identify candidate intercellular signaling changes after stroke while remaining inferential.

Further Downstream Characterization. Transcription factor activity inference, phenotype-associated program analysis, and in silico perturbation move downstream analyses from descriptive cell-state changes toward candidate regulators and disease-associated gene programs. Regulator activity inference using decoupleR suggested elevated Rela activity in microglia and fibroblasts at multiple time points after MCAO [132]. In macrophages, integrated hdWGCNA and pySCENIC analyses identified an inflammatory module associated with Mrc1^+^ macrophages and ischemic stroke, prioritizing Tnf as a hub gene and Stat3, Myc, and Nfil3 as candidate transcriptional regulators [133]. Phenotype-associated analysis prioritized endothelial cell subsets associated with stroke, and DoRothEA suggested subset-specific transcription factor activity patterns within these endothelial populations [46]. In silico knockout analyses predicted that perturbation of Ftl1, Fth1, or CLEC4D may induce immune response-related transcriptional reprogramming in microglia, macrophages, or neutrophils [134,135]. These approaches help prioritize candidate regulators and genes for further validation.

Therapeutic Intervention. These downstream analyses have also been extended to neuroprotective and therapeutic intervention studies. Olig2 mRNA delivery by lipid nanoparticles increased the relative abundance of oligodendrocytes [136], whereas trajectory analysis in a repetitive Transcranial Magnetic stimulation study suggested that treatment may promote the transition of pericytes toward vascular smooth muscle cells [137]. Cell type-specific analyses further localized treatment-associated molecular responses, including increased Hmgb1 and decreased Camk2n1 expression in astrocyte C2 subsets after low-intensity focused ultrasound [138] and prioritization of Nrf1 and Pou2f1 as candidate transcription factors in oligodendrocytes responsive to outer membrane vesicle-loaded pioglitazone treatment [139]. Functional enrichment analysis indicated activation of the BDNF pathway in inhibitory neurons and Sv2c-expressing interneurons after pretreatment of adeno-associated virus VEGF-C intracerebrospinal delivery [140]. At the intercellular level, cell–cell communication analysis predicted that quercetin-loaded extracellular vesicles might increase neuron–oligodendrocyte communication and decrease neuron–immune cell communication after stroke [141]. Together, these intervention-related applications suggest that sc/snRNA-seq analyses can help evaluate how candidate therapies reshape cell populations, cell states, molecular programs, and potential intercellular signaling networks in the ischemic brain.

**Table 1:** Downstream Bioinformatic Analyses and Their Applications in Ischemic Stroke Research.

Downstream Analysis	Applications in Stroke Research (Cellular and Molecular Mechanisms)
Cell Clustering and Annotation	Identification and annotation of major cell types and heterogeneous cell subsets, including glial, neuronal, immune, and vascular cell populations [23]
Cellular Composition and Differential Abundance	Assessment of post-stroke changes in cell-type and subset composition in untreated stroke, including immune and inflammatory cells [23,107], neurovascular unit/BBB-associated and angiogenesis-related cells [23,104], neurons [105] and NSCs [106], as well as treatment-associated compositional changes [136]
Trajectory and Cell Fate Inference	Inference of cell-state transitions and differentiation trajectories, including microglial reprogramming [128,129], neutrophil aging-associated reprogramming [130], NSC differentiation [27], and treatment-associated cell-state transitions [137]
Cell–Cell Communication	Inference of predicted intercellular communication after stroke in untreated conditions, including microglia-associated [23] and vascular-related signaling [131], as well as treatment-associated communication changes [141]
Differential Gene Expression	Identification of cell type-specific genes and transcriptional changes after stroke, including inflammation-related genes [108,109], neurovascular unit/BBB-associated and angiogenesis-related changes [110,111], metabolic processes [112], aging-associated changes [107], regeneration-related genes [111], and treatment-associated responses [138]
Functional Enrichment	Identification of enriched biological pathways after stroke, including immune responses [23], neurovascular unit/BBB-associated and angiogenesis-related pathways [104,111], cell death [114], metabolic pathways [115], and treatment-associated pathways [140]
Gene Set Scoring	Evaluation of predefined biological signatures after stroke, including NET-related scores [53], ferroptosis [116], autophagy [117], cuproptosis [118], disulfidptosis [119], pyroptosis and necroptosis scores [120], glycolysis, oxidative phosphorylation, and oxidative stress [121,122,123], cellular senescence [124], and SASP [125]
Metabolic Pathway Inference	Assessment of metabolic pathway activity or flux, including fatty acid metabolism [126] and amino acid uptake [127]
Transcription Factor Regulatory Network	Identification of candidate transcriptional regulators and regulatory networks in untreated stroke, including inflammation-related regulation [132] and treatment-associated regulatory responses [139]
Phenotype-Associated Program and Module	Identification of phenotype-associated gene programs and modules, including inflammation-related modules [133] and stroke-associated endothelial cell subsets [46]
In Silico Perturbation	Simulation of the effects of gene knockout involving Ftl1, Fth1, and CLEC4D on immune cells [134,135]

## 6. Single-Cell Transcriptomics in Ischemic Stroke Beyond the Brain

In previous studies, the application of single-cell transcriptomics in ischemic stroke has predominantly focused on elucidating cellular heterogeneity and local pathophysiological processes within the brain parenchyma. However, in recent years, this brain-centric paradigm has progressively expanded to a wide range of additional biological contexts.

At the spatial level, single-cell approaches have been increasingly applied to extra-cerebral tissues and cross-organ systems, including peripheral blood [142], thrombi [143], meningeal lymphatics [144], skull bone marrow [145], spleen [146], gut [147], vasculature, such as the aorta [148] and atherosclerotic plaques [149], and the heart [150], thereby revealing systemic immune and inflammatory networks associated with stroke. Clinical studies have identified multiple factors that affect the risk and prognosis of stroke, such as abnormal sleep duration and metabolic disorders [151,152]. At the level of biological modifiers, single-cell transcriptomics has been utilized to dissect the influence of diverse factors on stroke pathophysiology, including sex differences [153], circadian rhythms [154], and distinct etiological or clinical contexts (e.g., perioperative stroke) [155]. Furthermore, this technology has been extended to investigate stroke-associated complications and long-term sequelae. For example, single-cell analyses have provided mechanistic insights into post-stroke depression [156], post-stroke cognitive impairment [157], epilepsy [158], and ischemic injury in other neural tissues such as the retina [159].

Collectively, these advances indicate that single-cell transcriptomics is evolving from a tool primarily used for localized lesion analysis into a powerful platform for dissecting the multi-organ, multi-factorial, and whole-course regulatory networks underlying ischemic stroke.

## 7. Integrative Analytical Applications of Single-Cell Transcriptomics

Beyond analyses based on individual datasets, single-cell transcriptomics in ischemic stroke can be employed in conjunction with other data modalities. Existing studies generally reflect three common integrative analytical strategies. First, scRNA-seq datasets derived from independent studies or publicly available resources can be analyzed using a unified research framework. Such analyses may involve datasets from the same tissue across multiple studies [132,133,160] or extend to coordinated investigations across different tissues or sample sources, such as the brain and peripheral blood [146,161]. It should be noted that such integration does not necessarily require direct merging of datasets at the data level but may also be implemented at the analytical design level within a single study. Second, single-cell transcriptomic data can be used in combination with bulk RNA sequencing data, enabling analyses that incorporate both cell-resolved and tissue-level transcriptional information in the same study [116,125,160,162,163]. Third, single-cell transcriptomics can be integrated with other omics data or analytical approaches, including epigenomic profiling (e.g., ATAC-seq) [164], genetic epidemiological methods such as Mendelian randomization [165,166], and other multi-omics data types. For example, a recent study integrated single-cell and bulk expression quantitative trait locus data with genome-wide association studies for stroke and its subtypes using summary-data-based Mendelian randomization and colocalization analyses to prioritize putative cell-type-specific causal genes. Subsequent analysis of an ischemic stroke scRNA-seq dataset showed increased Lipa expression in mouse stroke samples, providing cell-resolved expression support for one of the prioritized genes [167]. These approaches extend the analytical scope beyond the transcriptomic layer and have been applied in several stroke-related studies.

## 8. Temporal and Spatial Resolution of sc/snRNA-Seq

sc/snRNA-seq technologies provide powerful tools for dissecting biological processes with unprecedented resolution. However, leveraging their full temporal and spatial potential requires distinct methodological approaches.

Temporal resolution in sc/snRNA seq is achievable, not inherently, but through strategic experimental design [168]. By sampling at different time points, such as during the acute, subacute, and chronic phases of ischemic stroke, researchers can reconstruct the progression of cellular and molecular responses. Although sc/snRNA-seq captures only a static snapshot at each timepoint, longitudinal experimental designs allow comparative analyses across defined intervals. To date, 23 studies have applied sc/snRNA-seq at two or more time points in MCAO models [53,105,114,121,131,142,169,170,171,172,173,174,175,176,177,178,179,180,181,182,183,184,185], with the longest experimental window from day 2 to day 21 post-stroke [178]. Characterizing the temporal evolution of cell states is essential for defining the Time Window (the period when reperfusion is most beneficial) [186]. Time-resolved single-cell analyses can thus identify optimal intervention timings and predict outcomes.

Despite these efforts, the destructive nature of sc/snRNA-seq limits the continuous tracking of cell state transitions within the same biological sample [187]. To overcome this limitation, several temporal transcriptomic strategies have been developed. Zman-seq introduces in vivo temporal “timestamps” into immune cells, enabling retrospective tracking of their transcriptional dynamics [187]. Complementary techniques, such as single-cell nanobiopsy and Live-seq, allow repeated transcriptomic profiling from living cells while preserving viability and functions [188,189]. As technological innovations advance, the possibility of achieving true time-resolved single-cell transcriptomics in post-stroke blood and brain tissues is becoming a practical reality.

Likewise, spatial resolution is not preserved in conventional sc/snRNA-seq due to the disruption of tissue architecture during dissociation or nuclear isolation. Although cell identities and transcriptomic signatures are well identified, their original spatial context within the brain remains unclear. To overcome this limitation, spatial transcriptomics (ST) platforms, including 10× Visium [190], Slide-seq [191], Stereo-seq [192], MERFISH [193], and Xenium [194], have been developed to retain and map spatial information. These tools are now increasingly integrated with sc/snRNA-seq to construct a more holistic spatiotemporal landscape of tissue biology [195].

In the context of stroke, ST has been primarily utilized to define ischemic territories, such as the infarct core and surrounding penumbra. Some studies have employed direct morphological evaluation to localize ischemic regions for ST analysis [178,196], while others have identified infarct zones based on spatial clusters showing marked downregulation of neuronal gene expression [121]. Reference-based integration has been performed, involving (1) clustering and annotating ST data from healthy brain samples using the Allen Brain Atlas, (2) integrating these annotated stroke datasets with unannotated stroke datasets for reclustering, and (3) using spatial coordinates to distinguish the infarct core from peri-infarct subregions [182,197]. Spatial transcriptomics directly informs the Tissue Window by assessing the viability and salvage potential of the ischemic penumbra after ischemia [186]. ST also enables the characterization of region-specific cellular composition, gene expression patterns, and cell–cell interaction networks within defined anatomical contexts [160,184,196,198,199].

In addition, recent studies have incorporated multiple time-point experimental designs into spatial transcriptomics, advancing toward true spatiotemporal single-cell profiling. These analyses reveal not only temporal dynamics but also location-dependent heterogeneity in cellular composition and gene expression, facilitating further investigation of stroke-related pathophysiological processes [178,182]. It is worth noting that the high-throughput molecular expression data generated by sc/snRNA seq and ST are naturally suitable for exploring emerging “molecular windows”—by systematically screening molecular markers at the transcriptome level, providing unique advantages for identifying salvageable ischemic tissues and providing outcome-oriented treatment strategies [200].

## 9. Discussion

**Figure 4:**
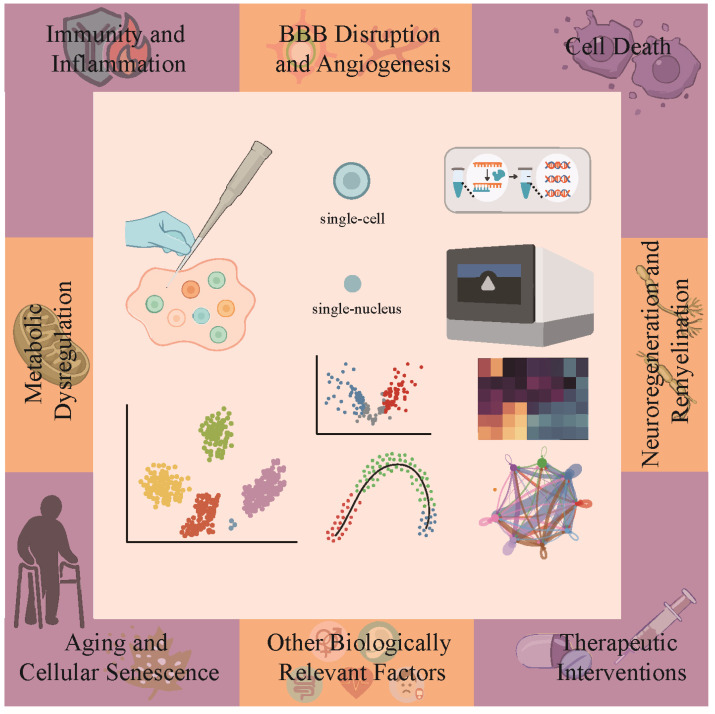
Overview of Ischemic Stroke Pathophysiology Revealed by sc/snRNA-seq.

The application of sc/snRNA-seq technologies has ushered in a new era in ischemic stroke research, offering unprecedented resolution to dissect cellular heterogeneity and pathophysiological complexity. Initially applied for the generation of brain cell atlases, sc/snRNA has significantly expanded in scope to investigate diverse mechanisms, including immune responses, transcriptional alterations in the neurovascular unit and blood–brain barrier-associated cells, cell death, metabolic dysregulation, aging, neuroregeneration, remyelination, and other biologically relevant processes and therapeutic interventions (Figure 4). Moreover, its utility has extended beyond the ischemic core and penumbra to encompass extracerebral organs and systems, comorbid conditions, and secondary complications, emphasizing its transformative potential for identifying novel therapeutic targets in ischemic stroke patients.

In addition to cell-type resolution, sc/snRNA-seq offers powerful insights into the temporal and spatial dynamics of stroke pathologies. Temporal resolution, achieved by sampling through strategic multi-time point sampling from the hyperacute to chronic phases, allows researchers to map transcriptomic changes and cell-state transitions in immune activity and glial remodeling. Although not an intrinsic feature of the technology, temporal insight is made possible by longitudinal experimental designs. Conversely, spatial resolution is lost during the tissue dissociation and nuclear isolation required for conventional sc/snRNA-seq. However, this limitation is increasingly addressed through the integration of spatial transcriptomics platforms, such as MERFISH, Slide-seq, and 10× Visium, which retain the anatomical context. This combination allows for the spatial localization of transcriptionally defined cell types, increasing our understanding of region-specific processes, such as peri-infarct inflammation, glial scar formation, and cortical remodeling. To maximize the scientific and translational impact of sc/snRNA-seq in ischemic stroke research, several strategic priorities should be prioritized. First, the integration of single-cell and single-nucleus datasets will offer a more comprehensive view of neurons, glia, and immune cells, especially given the challenges of capturing neuronal populations using scRNA-seq alone. Second, the experimental design must be problem-driven, focusing on clinically relevant issues such as futile recanalization [201], hemorrhagic transformation, recurrent stroke [202], or region-specific vulnerability of structures such as the thalamus or prefrontal cortex. Third, adopting multi-timepoint, multi-omics, and cross-species strategies, including non-human primate models, will enhance the biological depth and translational potential of these studies. Functional validation of key transcriptional targets and pathways remains essential to ensure biological relevance. Equally important is the expansion of sc/snRNA-seq into clinical research through the analysis of accessible human biospecimens, such as blood, thrombi, cerebrospinal fluid, and brain tissue, to identify conserved biomarkers and intervention points across species.

Looking ahead, the continued evolution of sc/snRNA-seq must go beyond descriptive cellular atlases to resolve the causal mechanisms underlying the pathophysiology and recovery of ischemic stroke. Embedding single-cell technologies within a hypothesis-driven framework, supported by functional validation and mechanistic interrogation, will allow researchers to progress from observational correlations to actionable insights. This shift is critical for translating single-cell discoveries into robust, targeted interventions for patients with ischemic stroke in clinical practice.

## 10. Conclusions

Taken together, this review systematically summarizes the application strategies and analytical approaches of single-cell and single-nucleus transcriptomics in ischemic stroke research. These applications span multiple dimensions, including the investigation of brain tissue pathology, extension to multi-organ and context-specific biological factors, and integration with diverse data types and multi-omics approaches. Moreover, advances in temporal and spatial resolution have further expanded the scope of these applications, enabling analyses across different biological dimensions. Collectively, single-cell transcriptomics provides a multi-level analytical framework for studying ischemic stroke. Future studies may benefit from more standardized experimental designs and coordinated application of different analytical strategies, facilitating systematic investigations in this field.

## Data Availability

No new data were created or analyzed in this study. Data sharing is not applicable to this article.

## Abbreviations

The following abbreviations are used in this manuscript:

BBB	Blood–brain barrier
CCA	Canonical correlation analysis
CCC	Cell–cell communication
CLEC4D	C-type lectin domain family 4 member D
CNS	Central nervous system
DE	Differential expression
DEGs	Differentially expressed genes
Fth1	Ferritin heavy polypeptide 1
Ftl1	Ferritin light polypeptide 1
GO	Gene Ontology
GSEA	Gene set enrichment analysis
I/R	Ischemia/reperfusion
KEGG	Kyoto Encyclopedia of Genes and Genomes
NET	Neutrophil extracellular trap
NSC	Neural stem cell
ORA	Over-representation analysis
PCA	Principal component analysis
SASP	Senescence-associated secretory phenotype
Sc/snRNA-seq	Single-cell and single-nucleus RNA sequencing
ScRNA-seq	Single-cell RNA sequencing
SnRNA-seq	Single-nucleus RNA sequencing
ST	Spatial transcriptomics
TF	Transcription factor
UMI	Unique molecular identifier
